# Teenagers and Young Adults with Cancer: An Exploration of Factors Contributing to Treatment Adherence

**DOI:** 10.1177/1043454221992302

**Published:** 2021-03-26

**Authors:** Elizabeth Carr, Leah Rosengarten

**Affiliations:** 1 Safari Day Unit, 156793 Lincoln County Hospital, Lincoln, UK; 2 Faculty of Health and Life Sciences, 5995 Northumbria University, Newcastle upon Tyne, UK

**Keywords:** adherence, teenagers and young adults, oncology

## Abstract

This systematic appraisal explores the literature surrounding treatment adherence in teenagers and young adults (TYAs) with cancer, with the aim of identifying influential factors that could affect adherence rates. This area is particularly important due to the increased risk of relapse and death associated with nonadherent behavior. In addition, TYAs are found to be the age group least likely to adhere to medical regimes. A comprehensive review of the literature was conducted and seven studies met the inclusion criteria, the articles were then critiqued using a data extraction form and eight themes were generated and discussed. This review highlights the complexities and difficulties in measuring adherence, as well as the key factors affecting adherence, before identifying implications for practice. Good communication and relationships are crucial between all parties involved in TYAs’ care including the patients, professionals, parents, and peers. A model of adherence was adapted on the basis of the result of the systematic review, other literature pertaining to adherence in TYAs, and the clinical experience of the authors. Personal factors and external factors, along with treatment factors and interactions with the system all have an effect on the patient's response or ability to adhere. It is apparent that there is a need for more high-quality qualitative and quantitative research in this area, with an emphasis on finding interventions that directly improve adherence specific to this age group.

## Introduction and Background

Cancer is the leading cause of nonaccidental death in adolescents, with research significantly suggesting that between 50% and 63% of patients do not adhere to their treatment (Kennard et al., 2004; Kondryn et al., 2011; Teenage Cancer Trust, 2014). Whilst even the most adherent patient can relapse or not survive depending on the type and severity of cancer they have, being adherent gives the best possible chance of success, especially concerning the use of chemotherapies and antibiotics (Kondryn et al., 2009; Rohan et al., 2013).

The World Health Organisation (2003, p. 13) defines adherence as “the degree to which a person's behaviour corresponds with the agreed recommendations from their health care provider.” There are overlapping definitions and interchangeable use of terms such as “adherence,” “compliance,” and “concordance” being used, but results reveal “adherence” as the most commonly used term in recent and current literature. The age range of patients focussed on within this systematic appraisal is between 13 and 24 years old and will consider “teenagers and young adults” (TYAs), as this is found to be the age group least likely to adhere to medical regimes (Abrams et al., 2007; Hawwa et al., 2009). Although this article aims to explore all aspects of nonadherence (NA), current research predominantly measures adherence in terms of medication taking.

There are a variety of different types of NA in medication taking; one is a clear refusal to take the prescribed medication (Nevins, 2002), where another may come from a patient accidentally missing a dose, realizing that there is no immediate medical consequence and then adopting a more casual attitude to medication taking (Abrams et al., 2007; Buchanan et al., 2014; Nevins, 2002). If there is no immediate effect or obvious repercussions to missing medication, this may cause the TYAs to doubt medical advice about the damaging effects of missing medication (Buchanan et al., 2014) and may even give the teenager a sense of invincibility and invulnerability (Dobbels et al., 2005). A further type of NA may occur if TYAs decide that the overall consequences of taking their medication, for example the medication being too strong or having too many side effects, are simply unacceptable and either omit or reduce the intake of the drug (Nevins, 2002; Windebank & Spinetta, 2008). It is noted that NA is not always intentional, but may occur due to confusion over dosage or who is responsible for the medication administration (Abrams et al., 2007). It is also important to remember that NA is not just about medication taking, it can also be in relation to not reporting illnesses or episodes such as temperatures that may affect treatment or need for treatment (Kondryn et al., 2009).

Previous research has highlighted that the adherence of TYAs with cancer is an area requiring further development and exploration (Enskar & Von Essen, 2007; Kondryn et al., 2011). It is established that there are links with nonadherent TYAs and higher rates of depression, lower self-esteem, and reports of poorer parent–child relationships (Kennard et al., 2004). Numerous studies have found a correlation between family relationships and treatment adherence, suggesting that the role of the parent has a significant impact on the patient's adherence (Kennard et al., 2004; Kondryn et al., 2011; Windebank & Spinetta, 2008). Additionally, due to an increased availability of oral medications for cancer treatment, patients are now expected to take more responsibility for their medical regimes at home (Abrams et al., 2007; Kennard et al., 2004).

## Methods

The Wiley Online Library, Proquest, Cochrane Collaboration, and Northumbria University's NORA database were searched. Due to the overlapping definitions and the interchangeable use of terms such as adherence, compliance, and concordance being used within the literature, all terms were searched to represent different attributes of this complex concept (Table 1).

**Table 1.** Search Terms.

Population	Teenage Adolescent Young Adult
Condition/disease	Cancer Oncology
Health behavior	Medication adherence Adherence Non-adherence Compliance Concordance

Table 2 displays the inclusion and exclusion criteria applied to the search. Due to the introduction of the National Institute for Health and Clinical Excellence report “Children's and Young People's Improving Outcome's Guidance” in 2005 and a change in the way and where TYAs received their treatment, journals from 2005 onwards only, have been included. To ensure the quality of data, any non-peer-reviewed research journals were excluded. Research that did not focus primarily on adherence was excluded to reduce the number of confounding variables. As the reasons for NA or abandonment of treatment differ largely in developing countries, due to financial difficulties, transportation issues, and differing beliefs and cultures (Sitaresmi et al., 2010), only studies conducted within developed countries have been included.

**Table 2.** Table of Inclusion/Exclusion Criteria.

Inclusion	Exclusion
Peer-reviewed journals only	Non-peer-reviewed
Qualitative and quantitative studies	Systematic review on the subject area
Articles written from 2005 onwards	Articles dated from before 2005
Studies in the English language	Non-oncological papers
Articles with the primary focus being adherence in TYAs with cancer	Articles where the primary focus was not adherence
Articles written in developed countries	Articles written in developing countries
Focussed on age range 13–24 year olds	Studies focussing outside the specified age range
Primary and secondary research	

*Note*. TYAs = teenagers and young adults.

The search resulted in 147 articles, the inclusion/exclusion criteria were applied, narrowing the search down to 35 articles. From this abstracts were read and the duplicate and irrelevant articles were removed, this left a remaining five articles. Hand searching of reference lists from relevant journal articles was also carried out and a further two articles were identified. This resulted in a total of seven articles being selected for this systematic appraisal (Table 3). The databases were searched regularly to find any new research published but no new articles were found.

**Table 3.** Table of Selected Articles.

Study	Authors and date	Title	Country and setting	Study design	Participants	Key findings
1.	Malbasa et al. (2007)	Adolescent adherence to oral therapy for leukaemia: A focus group study	USA Children's Hospital in Cleveland	Secondary analysis of focus group data derived from a larger pilot study Qualitative	6 participants Convenience sample 2 male, 4 female Aged between 16 and 23 years	Through participant reports, four key themes arose that influenced adherence: normalcy, egocentricity, concrete thinking, and parental involvement
2.	Pai et al. (2008)	Correspondence between objective and subjective reports of adherence among adolescents with acute lymphoblastic leukaemia	USA Multi-site study— Cincinnati and Cleveland	Cluster analysis of self-reports of adherence and bioassays adherence indicators over 112 days Quantitative	51 adolescents Aged between 12 and 19 years	Both self-report and bioassays showed the prevalence of non-adherence. Adolescents who report problems early on are likely to continue with adherence issues throughout treatment
3.	Hawwa et al. (2009)	The development of an objective methodology to measure medication adherence to oral thiopurines in paediatric patients with acute lymphoblastic leukaemia—an exploratory study	United Kingdom Haematology and Oncology Outpatient Department, Children's Hospital	Parents/guardians to complete reports of adherence at each clinic visit, along with blood samples taken to determine the metabolite content Quantitative	19 patients aged between 3 and 17 years Convenience sample	Results showed that teenagers are significantly more nonadherent in both self-reports and metabolites than younger children
4.	Kondryn et al. (2009)	Treatment non-adherence in teenage and young adult cancer patients: A preliminary study of patient perceptions	United Kingdom TYA Oncology unit	Self-reporting questionnaires were used to report on adherence to treatment Quantitative	33 patients Aged 16–24 years Convenience sample	Participants' reports showed that they did not adhere to up to 38% of low-risk treatment. Twenty-five percent had not reported pyrexia and diarrhea. Thirty-three percent had considered stopping treatment.
5.	Stinson et al. (2012)	Disease self-management needs of adolescents with cancer: Perspectives of Adolescents with Cancer and their parents and healthcare provider	Canada Cancer Care Centre, Toronto	Descriptive design—using grounded theory Semi-structured individual interviews and focus group interviews Qualitative	29 TYAs—interviews and focus groups 33 parents—interviews and focus groups 22 health professionals—focus groups	Four themes emerged through participant reporting: Disease knowledge and cancer care skills, knowledge and skills to aid the transition, accessible healthcare services, and support for the TYAs. These strategies increase adherence.
6.	Rohan et al. (2013)	Electronic monitoring of medication adherence in early maintenance phase treatment for pediatric leukemia and lymphoma: Identifying patterns of non adherence.	USA Across six Paediatric Cancer Centres	Objective observational method Electronic monitoring of 6MP adherence Quantitative	139 patients aged 7–19 years	The mean adherence was 86.2%, below the 95% target. There were three different types of adherence highlighted: Exemplary, deteriorating, or chronically poor.
7.	Rohan et al. (2017)	Measuring medication adherence in pediatric cancer: An approach to validation.	USA Across six Paediatric Cancer Centres	Pharmacological and behavior adherence measures. secondary analysis of data. Combined blood level monitoring of 6MP with electronic medication monitoring. Quantitative	139 patients aged 7–19 years	Identified three groups from blood monitoring. One group had low levels of 6MP metabolites (40.8%) and two other groups had adequate adherence (59.2%). Patients with low metabolites had consistently lower behavioral adherence rates.

*Note*. TYAs = teenagers and young adults.

To establish themes from the literature, the six-phase guide to conducting a thematic analysis, as outlined by Braun and Clarke (2006), was used. Researchers initially independently familiarized themselves with the seven chosen articles and generated initial codes. These codes were then jointly reviewed by both researchers and discussed to identify interrelating themes. Only minor discrepancies were apparent during coding which mostly related to precise naming of the theme; these were discussed and names reflective of the wider literature were decided upon.

## Results

All studies found similar results despite the difference in countries and methods chosen. Eight themes were identified during the data analysis, these were difficulties in measuring adherence, healthcare professionals’ role in addressing adherence, interventions to improve adherence, the role of the parents, the transition to maintenance, more information for TYAs, risk-taking, and a desire for “normal life.” Table 4 identifies how each theme arose from each of the seven articles.

**Table 4.** Themes.

Themes	Study 1	Study 2	Study 3	Study 4	Study 5	Study 6	Study 7
Difficulties in measuring adherence	✓	✓	✓	✓	✓	✓	✓
Healthcare professional's role in addressing adherence	✓	✓	✓	✓	✓	✓	✓
Interventions to improve adherence	✓	✓	✓	✓	✓	✓	✓
Role of the parents	✓		✓	✓	✓	✓	✓
Transition to maintenance	✓	✓	✓			✓	✓
More information for TYAs	✓	✓				✓	✓
Risk–taking	✓			✓	✓		
Desire for “normal life”	✓			✓	✓		

*Note*. TYAs = teenagers and young adults.

### Difficulties in Measuring Adherence

The first theme identified from the thematic analysis was difficulties in measuring adherence, which was highlighted in all seven studies. The studies used a variety of methods to address and measure adherence including blood tests, electronic pillbox monitoring, and self-reporting both during treatment and retrospectively.

Studies 2, 3, 4, 6, and 7 directly measured adherence through a quantitative design method. Studies 2, 6, and 7 used a measure of 95% or above as being an optimum adherence rate, Bhatia et al. (2012) also recommend this percentage, with this in mind none of the studies measured adherence at above 95% adherence “The mean adherence rate was 86.2%. Adherence rates declined over the 1 month follow to 83.3%” (Study 6, p. 1).

Studies 2, 3, and 6 aimed to directly measure adherence using objective and subjective methods of measurement and reporting. As there is currently no unified approach, assessing adherence is difficult, which has been highlighted as a commonly discussed limitation (Studies 3, 6, and 7). It was identified in Bhatia et al.’s (2012) study that if adherence falls below 95%, the rate of relapse increased. Studies 2, 6, and 7 used this benchmark of 95% for the TYAs to be considered adherent. However, determining how to measure this percentage has been deemed problematic as patients, parents, and professionals' perspectives on what adherence is, may also differ (Studies 3, 6, and 7).

Studies 2, 4, and 7 used blood tests to record levels of adherence, whilst Study 2 also used subjective reports of adherence. The results from Study 4 showed that TYAs are significantly more likely to show nonadherent in both self-reports and metabolites than younger children “Demographic factors examined for possible association with non-adherence were gender and age…higher age was significantly associated with non-adherence” (Study 2, p. 1110). In contrast to this whilst Studies 6 and 7 found adherence was below the optimum level, they reported no difference in demographic characteristics such as age, ethnicity, race, and family structure.

Although, it is argued that using objective measurements such as blood tests and electronic monitoring, for example pillboxes, is a more reliable measurement than self-reporting, even these methods are subject to criticism. Patient manipulation and pharmacokinetic variability, for example drug absorption, distribution, and metabolism must be taken into consideration (Study 6). Studies 2 and 7, therefore, sought to combine these two measures to combine the benefit of both methods of monitoring.

Study 3 used a self-reporting questionnaire on adherence; they found TYAs did not adhere to up to 38% of low-risk treatment. Twenty-five percent had not reported pyrexia and diarrhea and 33% had considered stopping treatment. Studies 2 and 3 argue that to address NA, it must be dealt with from the beginning of treatment and continually reassessed; however, research suggests that often adherence is addressed retrospectively (Studies 1, 3, and 4). There are a lot of reservations in using self-reports of adherence due to their subjective nature often resulting in misrepresentation and overestimation of adherence (Study 3). Despite this self-reporting was found to be the most commonly used tool in measuring adherence, due to it being easy to do, inexpensive, and easily utilized by all members of the healthcare team (Studies 1, 3, and 4). The mixed-method approach used in Study 2 found a correlation between self-reporting and the blood level results, highlighting that when asked to report the TYAs reported truthfully about adherence. It was also found that TYAs who reported NA early on were likely to continue with adherence issues throughout treatment.

### Healthcare Professional's role in Addressing Adherence

The role of the healthcare professional in recognizing and addressing NA was apparent within all of the articles. “Findings of this investigation clearly illustrate the need for medical providers to repeatedly assess adherence” (Study 2, p. 234). This was also a recommendation in Study 3 where it was interestingly found that as the number of visits to the healthcare professional increased patients became more open in reporting NA. This may indicate patients need some time to report NA, thus supporting the need to continually monitor. TYAs should be asked whether they are taking their medications as prescribed and whether the regimen is causing them distress or side effects, then address any issues found. The results from the studies found this communication optimum in achieving adherence (Studies 1, 4, 5, and 7). This communication must be done in an open and nonstigmatized way, so that good patient–professional relationships are maintained. This building of trust and mutual information sharing constitutes what can be argued as being one of the most important intervention strategies that we can apply to reduce TYAs NA. It is important to remember that the responsibility does not solely lie with the medical team, as the nursing staff and other health professionals also have a responsibility in addressing NA.

### Interventions to Improve Adherence

Interventions aimed at promoting adherent behavior was also a theme arising from all of the articles. This is supported in the following quote “it was recognised that because of their development stage and shifting levels of responsibility between adolescents with cancer (AWC), their parents and healthcare team, assisting AWC in developing these skills is vital” (Study 5, p. 282). The suggested interventions based on the findings of the studies ranged from monitoring adherence to communication with healthcare professionals and other TYAs. It is consistently argued that early intervention is crucial in addressing adherence. However, due to the limited methodological approaches and small sample sizes used within the articles, there is a clear need for both focussed qualitative and quantitative research to be conducted to substantiate these findings further.

The first key recommendation is that simply monitoring adherence can increase adherence (Studies 1–3, 6, and 7). As the patients/parents within Studies 2, 3, 6, and 7 were aware that adherence was being monitored subsequently findings may have been influenced by patient and family expectations and reactivity. Study 6 argues that if TYAs are aware that adherence is being monitored either objectively or subjectively this could increase adherence. Yet despite this, the studies still found incidents of NA. Discussing the importance of adherence may be beneficial because it may spur those with poor adherence to improve and may encourage those with good adherence to continue (Studies 1 and 4). A positive and effective communication is therefore an important intervention in addressing adherence.

Communication with peers was also highlighted as an intervention in Studies 1, 4, and 5. Study 5 argues that establishing a connection with other TYAs with cancer is critical in supporting adolescent development and self-management skills. Therefore, this highlights that peer interaction may actually improve adherence rates.

### Role of the Parents

The role of the parents in TYAs NA was highlighted within six of the seven articles, with research suggesting that NA is highest when TYAs are responsible for their own medication (Studies 1 and 4). “Participants identified parental support as a key factor in their adherence to maintenance therapy” (Study 1, p. 147). It was highlighted as a main finding in four articles, thus showing strength in the importance of their involvement and the parent/child relationship.

Study 4 highlighted a correlation between family relationships and adherence, suggesting that the role of the parent significantly impacts on the patient's willingness or ability to adhere. Study 1 identified parental support as a key factor in achieving adherence, this was either through administering the medication or through motivating the TYAs to self-administer. Three out of four participants in this study who reported parental involvement were deemed to have “optimal” adherence. It was also found that mothers played a more significant role. Study 3 found similar results stating that TYAs were the worst compliers; this could be due to their managing their own medication administration. Even if the TYAs self-administers their medication, the parent still has a role in monitoring/encouraging this (Studies 1 and 3). It is important to remember that some TYAs have no choice in solely managing their treatment and some TYAs may be able to solely manage. However, results from Study 1 demonstrated that parental involvement/encouragement positively affected adherence.

### Transition to Maintenance

Studies 1, 2, 3, 6, and 7 focussed on acute lymphoblastic leukemia (ALL), the treatment for this is completed in several phases, which led to the 5th theme; transition to maintenance.

Once initial phases of treatment for ALL have been completed, usually lasting around 6 months the TYAs then starts the maintenance phase (Study 2). The maintenance phase is a prolonged course of oral chemotherapy 6-mercaptopurine (6MP) and is essential in achieving a long-term, disease-free survival (Study 3). However, it was found that this is when NA is at its highest. Study 1 found that many of their participants identified the transition to maintenance as a turning point in their treatment. Some viewed this as a positive turning point, expressing relief. However, some TYAs expressed difficulty in accepting that despite being leukemia-free they must continue this drug therapy. This could be due to most TYAs being asymptomatic during this phase (Studies 3 and 6). The length of the maintenance phase may also be a factor in relation to NA, the TYAs may become nonchalant about taking medication (Study 1). Study 6 found that adherence deteriorates over the course of treatment lasting ∼2 years.

Studies suggest that during this phase of treatment, more information should be given to the TYAs about the importance of adherence (Studies 3, 6, and 7).

### More Information for TYAs

With the above theme in mind, it was recommended in Studies 1, 2, and 7 that more information be given to TYAs regarding the maintenance phase. This information should remind TYAs that although they may be asymptomatic, the rate of relapse increases if adherence is not optimal (Studies 1 and 2). This recommendation for more information is not limited to only TYAs on maintenance regimes in ALL treatment, but can also be useful for all TYAs undergoing cancer treatment

Study 1 found that clinicians unknowingly reinforced NA, by giving positive feedback regarding their remission status. The participants drew a casual correlation between not taking their medication and continuing to get better. This could be due to an inability to conceptualize long-term consequences for NA (Study 1). Study 6 argues NA is found to increase the rate of relapse both during and post-treatment. Health professionals and parents must be aware of this and continue to encourage adherence even when patients are asymptomatic (Studies 1 and 6). However, a significant point to remember is that even the most adherent TYAs may relapse but optimum adherence gives the TYAs the best possible chance at long-term survival.

Study 5 focussed on improving TYAs self-management skills, these skills are found to increase adherence. The study highlighted the TYAs need for information surrounding immediate and long-term impacts, body image, and lifestyle choices. With this in mind, they propose that all TYAs with cancer are given the necessary information and education they require throughout treatment.

### Risk-Taking

Risk-taking was a theme that arose from Studies 1, 4, and 5. Though precisely what constitutes risk can be difficult to define, when relating this to TYAs NA, there is a life-endangering risk associated with not adhering to medication regimes.

Study 1 found what they referred to as “egocentricity” and “concrete thinking” had an effect on adherence. These led to risk-taking behavior, which was also mentioned in Studies 4 and 5. Whilst the studies suggest that risk-taking is a normal and adaptive part of adolescence, however, this type of risk-taking is potentially life-endangering.

Being asymptomatic was a recurring factor in displaying risk-taking behavior, participants in Study 1 reported that they were getting better regardless of whether they took their prescribed medication. Whilst the repercussions of withholding a few doses are not immediate the psychological effects are far greater. As the TYAs may then start to doubt what their doctors, other medical professionals, and parents are telling them about the damaging effects of missing medication (Study 1).

### A Desire for “Normal Life”

A desire to feel “normal” was highlighted within Studies 1, 4, and 5. These feelings of wanting to remain “normal” could be in relation to acknowledging their illness, family life, peer relations, and social activities (Studies 1 and 4). Study 1 reports that participants, once able to return to what they deemed “normal” activities, continued to take medication, which acted as a constant reminder that they remained different. Although this relationship is not mutually exclusive, the results of this study identified the need for normalcy in nearly all aspects of the patients' lives.

Studies 1 and 5 also place focus on improving quality of life (QOL) for TYAs with cancer. Study 5 aims to improve self-management skills in areas such as managing symptoms, lifestyle, treatment, physical and psychological consequences of living with cancer to improve QOL, along with adherence rates and outcome.

Poor QOL could be a result of numerous factors including dealing with a potentially life-threatening illness, changes in physical appearance, and emotional well-being. TYAs with a cancer diagnosis may already feel differently from their peers. The added changes in physical appearance were found to have various consequences on the TYAs emotional well-being, such as aggression, depression, low self-esteem, and feelings of emotional frustration towards their dependency on treatment (Studies 1 and 2). Study 1 suggests that these factors are closely linked to adherence rates. The self-management strategies in Study 5 aimed at improving these factors include increasing knowledge base, learning opportunities, and establishing meaningful social support networks (Study 5).

However, it is important to remember the relationship between QOL and adherence although linked is not causal in nature, improved QOL does not automatically improve adherence and vice versa. This is certainly an area for further research.

## Discussion

Currently, there is not one specific measurement of medication adherence for TYAs, as there are inconsistencies in the literature surrounding the best methods of measurement. The difficulties around measuring adherence come from divided opinion on whether objective or subjective measurements provide the best results.

Some studies have conducted blood tests to measure adherence which is often done to measure 6MP levels within the bloodstream (Hawwa et al., 2009; Pai et al., 2008; Rohan et al., 2017; Traore et al., 2006). Measuring adherence in this way can provide information that is less vulnerable to self-reporting bias (Pai et al., 2008). If TYAs have low levels of the 6MP metabolites, it would suggest that the TYAs are not receiving their full medication dosages, for example being nonadherent. However, pharmacokinetic variability must be considered in relation to drug absorption, distribution, and metabolism (Ruddy et al., 2009).

Another way of measuring adherence is through electronic monitoring, this approach consists of a system whereby the cap on a pill bottle electronically records the date and time the cap of the pill bottle is removed (Rohan et al., 2013; Ruddy et al., 2009). Yet this approach can be subject to patient manipulation as the pill may be discarded or not taken at the exact time the bottle is opened (Rohan et al., 2013, 2017; Ruddy et al., 2009). It has been demonstrated that combining blood level monitoring with electronic monitoring can provide more accurate information as to whether low 6MP metabolite levels are due to NA or pharmacokinetic variability (Rohan et al., 2017).

Subjective reports of adherence have also been a focus for research (Kondryn et al., 2009; Pai et al., 2008). Often subjective reports identifying nonadherent behavior are carried out retrospectively. However, Kondryn et al. (2009) and Pai et al. (2008) argue that to address NA, it must be dealt with from the beginning of treatment and continually reassessed. There is a lot of reservations in using self-reports of adherence due to the subjective nature often resulting in misrepresentation and overestimation of adherence (Hawwa et al., 2009). Using self-reports is the most widely utilized as it is inexpensive, easy to use, and can be carried out by any member of the healthcare team (Hawwa et al., 2009; Malbasa et al., 2007).

Rohan et al. (2013) and Ruddy et al. (2009) argue that if TYAs are aware that adherence is being monitored either objectively or subjectively this could increase adherence, known as “the Hawthorn effect” (Ruddy et al., 2009). This would perhaps indicate that the method of measuring adherence may never be fully reflective of actual NA. However, this may also support the use of monitoring of NA in TYAs to improve adherence.

The “healthcare professional's role in addressing adherence” was a theme identified. Numerous studies have highlighted the importance of their role in addressing adherence (Kondryn et al., 2009; Malbasa et al., 2007; Rohan et al., 2017; Ruddy et al., 2009). The role of healthcare professionals in ensuring good relationships and a positive interaction with the healthcare system is complex, with healthcare professionals being required to build trust and have open communication with TYAs, whilst also monitoring for NA and providing education.

One key role of the healthcare professional is to monitor NA by asking TYAs whether they are taking their medications as prescribed and whether their regime is causing them distress or problems with side effects (Ruddy et al., 2009; Windebank & Spinetta, 2008) However, this communication must be carried out in an open and nonstigmatizing way, so that good patient–professional relationships are maintained (Windebank & Spinetta, 2008).

Ruddy et al. (2009) and Stinson et al. (2012) argue that discussing the importance of adherence has also been found to be beneficial, as it may spur those with poor adherence to improve and may encourage those with good adherence to continue. Along with this, they stated that TYAs must be educated in their treatment regimes, to fully understand what they are taking and why (Coyne et al., 2019; Ruddy et al., 2009; Stinson et al., 2012). The National Service Framework for Children (Department of Health, 2004) advocates this shared decision-making between parents or carers, children or young people, and professionals. This is achieved by involving TYAs in discussions of the risks and benefits of treatment and taking into account their values and beliefs and the effects the proposed treatment could have on their daily living. This increased collaboration has also been recognized within government initiatives such as “Transforming Participation in Health and Care” (NHS, 2013) and “Patient and Public Involvement in the NHS” (Great Britain. Parliament. House of Common, 2007).

Whilst the term “adherence” is most commonly used within the literature, the term “concordance” is becoming a more popular concept. This is where the prescriber and the patient come to an agreement about their treatment regime (Horne et al., 2005). The active inclusion of the TYAs in this dialogue regarding treatment helps set an atmosphere of compliance. It can improve the professional–patient relationship from compliance (doctor's recommendations) through to adherence (sticking to the plan) to concordance (harmonizing) (Coyne et al., 2019; Horne et al., 2005; Windebank & Spinetta, 2008).

Consideration of the “Hawthorn effect” (Rohan et al., 2013; Ruddy et al., 2009) again has a role to play here as healthcare systems that place an importance on monitoring adherence, could see the benefits of improved adherence. However, this action must be offset against the risk that monitoring adherence could endanger the quality of the trusting relationships that healthcare professionals seek to build with TYAs.

The role of the parents was another theme highlighted in the appraisal. Numerous studies have found a correlation between family relationships and compliance, suggesting that the role of the parent significantly impacts the patient's adherence (Kennard et al., 2004; Kondryn et al., 2011; Windebank & Spinetta, 2008). It is found that inconsistent styles of parenting and lack of ability to discipline reinforce unwanted behavior such as NA (Kalb & Loeber, 2003). There are numerous factors that influence parenting style, which in turn impact rates of adherence, for example when anxious parents restrict their TYAs behavior unnecessarily, it was found to negatively influence adherence (Kennard et al., 2004).

Berquist et al. (2008) found that adolescents from a single-parent family are at a higher risk of NA, this could be down to a variety of reasons. It could be linked to socio-economic status, as single-parent families on average have a lower income, due to an inability to work many hours or unemployment due to the needs of having an ill child (Brown et al., 2008; Lubkin & Larsen, 2013; Windebank & Spinetta, 2008). Or having to work many hours to independently support the family and as a result not being able to attend appointments or have the time to manage or monitor medication intake. Studies have found that patients whose parents accompanied them to appointments were significantly more likely to adhere to medical treatments (Griffin & Elkin, 2001). This could affect the adolescent as Griffin and Elkin (2001) reported that feelings of resentment and hostility from the parents often affected adherence and the parent–child relationship.

An important developmental task during adolescence is forming an identity separate from your parents, it is also a time for growing independence (Buchanan et al., 2014; Grinyer, 2009) yet it was found that one of the most common reasons teenagers miss medication is that they simply forget. Research suggests that despite a growth in maturity, teenagers lack the ability to imagine abstract concepts, for example the future consequences of present actions (Buchanan et al., 2014; Windebank & Spinetta, 2008).

Whilst the findings from the appraisal identified transition to maintenance as a key time for NA, Grinyer (2009) states that one of the most common reasons for not taking or reducing medication intake is down to the side effects. This is an important consideration in TYAs NA, as the principles of medical ethics guide that the benefits of a medication should outweigh the harm, and this should allow for consideration of the individual's perspective. More information for TYAs needs to be given with regard to all stages of treatment both in the initial stages and in the maintenance phase to better prepare TYAs for their cancer journey and issues that may arise. This information is also important for healthcare professionals to have a better understanding of how the TYAs may be feeling and why they may be NA.

Some of the most commonly reported side effects of cancer treatment are hair loss, weight gain or loss, mucositis (inflammation and ulceration of the mucus membranes), pain, and lethargy (Abrams et al., 2007; Grinyer, 2009; Miller et al., 2011). Whilst undergoing treatment, TYAs frequently express concerns about appearance and body image. Whilst weight gain and acne are common amongst teenagers regardless of health (NHS, 2012), the effects of this are likely to be accelerated and intensified when on medication such as steroids (Anderzen-Carlsson et al., 2012). One study found that by the end of treatment for ALL 23% of the patients were obese, compared with only 14% on diagnosis (Withycombe et al., 2009). TYAs may reduce or omit their medication in an attempt to avoid gaining extra/unwanted weight. Grinyer (2009, p. 204) found that weight gain during treatment was described as “distressing and demoralizing.” As a result of this the TYAs lost confidence and self-esteem, Kennard et al. (2004) suggest that there are close links between higher self-esteem and higher adherence rates.

As nausea and sickness are some of the most commonly reported side effects of chemotherapy, weight loss can also affect the TYAs making them feel self-conscious about their appearance. Again, they may, therefore, decide to omit their medication (Windebank & Spinetta, 2008). Whilst there is a vast range of antiemetic therapies available patients may refuse or forget to take them, they may also feel that they do not work so omit to take them (Horne et al., 2005; Windebank & Spinetta, 2008).

Grinyer (2009) reported in her study that hair loss was the biggest cause of anxiety in TYAs with cancer. Hair can be lost due to chemotherapy or in preparation for surgery, for example for a brain tumor. Patients are likely to find this distressing (Hedstrom et al., 2004; Gibson & Soanes, 2008). Patients in this study reported wanting to delay treatment to have their hair for longer. Anderzen-Carlsson et al. (2012) found similar results, stating that the girls in their study all reported difficulty in accepting hair loss, and many faced denial about it happening to them.

TYAs may also have scars from tumor resections or from having central venous access. Surprisingly, little research has been done in relation to scars and their impact on QOL (Brown et al., 2008); however, research does suggest that scars do have an impact on self-consciousness and anxiety (Abrams et al., 2007; Tebble et al., 2005). Scars can have an impact on communication skills, relationships, and leisure activities (Brown et al., 2008). Along with an increasingly peer focussed lifestyle and the natural changes in the body and body consciousness during adolescents, it could be argued that the addition of scarring can be detrimental to a patient's mental health (Michaud et al., 2004; Abrams et al., 2007).

According to De Castro and Moreno-Jimenez (2008), body consciousness and issues around physical appearance could also be due to TYAs having to show their bodies to medical professionals on a regular basis, these experiences are reported as shaming and compromising the patient's privacy and negativity affecting their relationship with their body. In response to this TYAs may downplay or chose not to disclose any issues/symptoms to the medical professional (Smith et al., 2007). It could be argued that this is also a type of NA, as it could result in a mismanagement of symptoms or even relapse (Smith et al., 2007; Windebank & Spinetta, 2008).

Studies suggest that hair loss, weight changes, the presence of a central venous catheter or a nasogastric tube, and scars from surgery, are the most disturbing physical changes, and can be devastating to an adolescent's self-image (Larouche & Chin-Peuckert, 2006; Lee et al., 2012). TYAs with a cancer diagnosis may already feel different from their peers, these added changes in physical appearance may have various consequences such as aggression, depression, low self-esteem, and feelings of emotional frustration towards their dependency on treatment (Abrams et al., 2007; Grinyer, 2009; Stinson et al., 2012).

To mitigate the possible damage of NA, the role of the healthcare professional is again paramount. It is argued that discussing the importance of adherence has also been found to be beneficial, as it may spur those with poor adherence to improve and may encourage those with good adherence to continue. Through educating TYAs in their treatment regimes, TYAs will be supported to fully understand why the benefits of their treatment outweigh the perceived harm (Coyne et al., 2019; Ruddy et al., 2009; Stinson et al., 2012).

Anxiety, low self-esteem, and social withdrawal are all psychological factors associated with cancer, and they may influence the adolescent's ability to understand the consequences of NA (Buchanan et al., 2014; Stinson et al., 2012). Strong links have been found between low self-esteem and low adherence rates, with studies suggesting the two are closely correlated (Abrams et al., 2007; Kennard et al., 2004).

Interestingly, scientific studies have found that embarrassment and self-consciousness in teenagers stimulate the same part of the brain as actual physical pain (Phillips, 2007). To prevent this it is possible that teenagers could omit to take their medication in an attempt to feel “normal” or “ordinary,” an example of this could be that they do not take their medication at the correct time so they do not have to interrupt social activities (Grinyer, 2009; Stinson et al., 2012). It is important to understand the challenges faced by adolescents to incorporate this complex and strict routine into an increasingly peer focussed lifestyle (Zelikovsky et al., 2008). Feinstein et al. (2005) found that although teenagers were aware they remained different from their peers, they made a continual effort to lead a “normal” life. These issues can then bring on anxiety and sometimes denial. Often denial can then serve as a defense mechanism against the anxiety and depression associated with their conditions (Feinstein et al., 2005).

Whilst it can be argued that many TYAs may attempt to lead a “normal” life, Griffin and Elkin (2001) found problems in social adjustment and withdrawal from their social groups. Numerous studies found that TYAs feel withdrawn from their peers during treatment (Grinyer, 2009; Sayer et al., 2007). This could be due to feelings of self-consciousness about appearance, or feelings of isolation and exclusion from social activities due to long periods spent in hospital and low school attendance (Griffin & Elkin, 2001; Michaud et al., 2004; Pini et al., 2013). This again highlights the importance of Teenage Cancer Trust (TCT) units and peer support.

Griffin and Elkin (2001), in their review of the current literature surrounding organ transplantation, found reports of problems in TYAs social adjustment and withdrawal from their social group. This was found to be much higher than the normative control sample of “well” children. Grinyer (2009) and Kennard et al. (2004) also found similar reports of social withdrawal. As adolescence is a time of psychosocial development, peer group acceptance is fundamental in the transition to adulthood. This lack of peer group support may prevent the TYAs from having a sense of belonging (Smith et al., 2007). Numerous studies have found that TYAs feel withdrawn from their peers during treatment (Grinyer, 2009; Sayer et al., 2007). This could be due to feelings of self-consciousness about appearance, or feelings of isolation and exclusion from social activities due to long periods spent in hospital, poor educational attainment, and low school attendance (Griffin & Elkin, 2001; Michaud et al., 2004; Pini et al., 2013).

Griffin and Elkin (2001) and Michaud et al. (2004) found that a TYA speaking to a peer who has had issues with adherence and becomes critically ill because of this may help the adolescent realize the life-threatening danger they are putting themselves in. There is little literature to support whether an intervention such as this would have a long-term effect on TYAs NA, although public health literature would argue that more factors are required for behavior change. For example, Kok et al. (2014) suggest that an individual requires a belief that their behavior may be a risk, a model for the desired behavior (such as the peer identified above), and a belief in the change.

Lastly, development theorists portray adolescence as a time for growth in maturity, the development of identity, new body image, and sexuality (Epleman, 2013; Lightfoot et al., 2013). Many studies suggest that risk-taking is a normal and adaptive part of adolescence (Kennard et al., 2004; Kondryn et al., 2011; Trevino et al., 2013). Whilst risk-taking can be seen as age-appropriate, the life-endangering risk of NA could be related to feelings of injustice and lack of control in an extremely strict and controlled lifestyle (Grinyer, 2009; Windebank & Spinetta, 2008). It could be argued that this risk-taking is similar to, or a form of self-harm, due to feelings of lack of control or in an attempt to seek attention. By identifying TYAs who demonstrate negative health beliefs or struggle emotionally with their condition, it may be possible to predict those TYAs who require additional support.

### Model of Adherence

**Figure 1.**
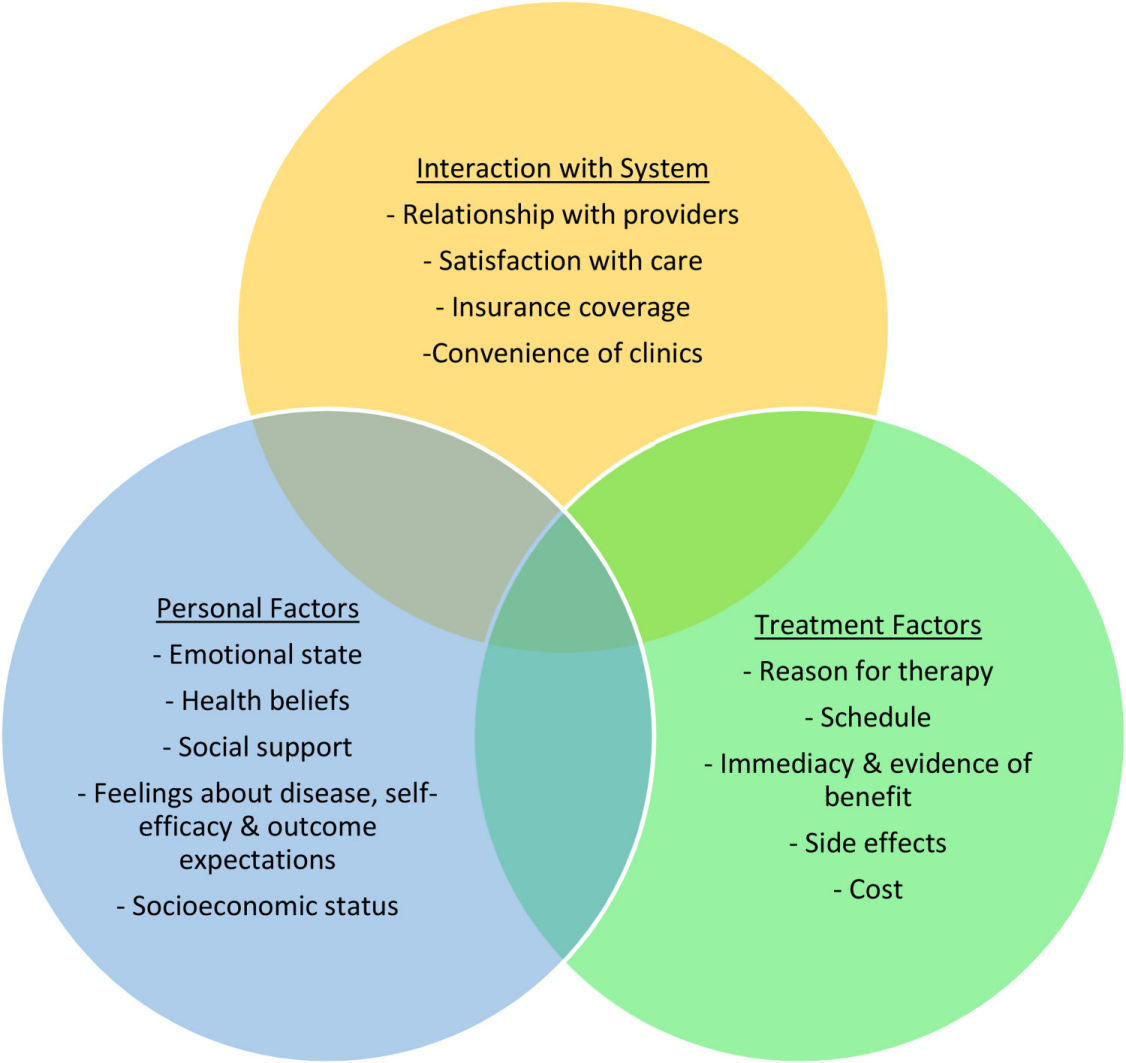
Model of adherence and persistence.

To address NA in TYAs, it is useful to consider the causative factors of NA, to help recognize those most at risk. Figure 1 shows Ruddy et al.’s (2009) bio-psychosocial model of adherence, which highlights the complexity of the phenomenon of adherence. This model is of interest as the themes identified in the appraisal can be fit into some of the different aspects of the model. However, the most obvious criticism of this model when applied to TYAs with cancer is that the role of the parents is not recognized but the literature identifies that this is an important factor in influencing adherence in TYAs (Kennard et al., 2004; Kondryn et al., 2011; Windebank & Spinetta, 2008).

### Implications for Practice

**Figure 2.**
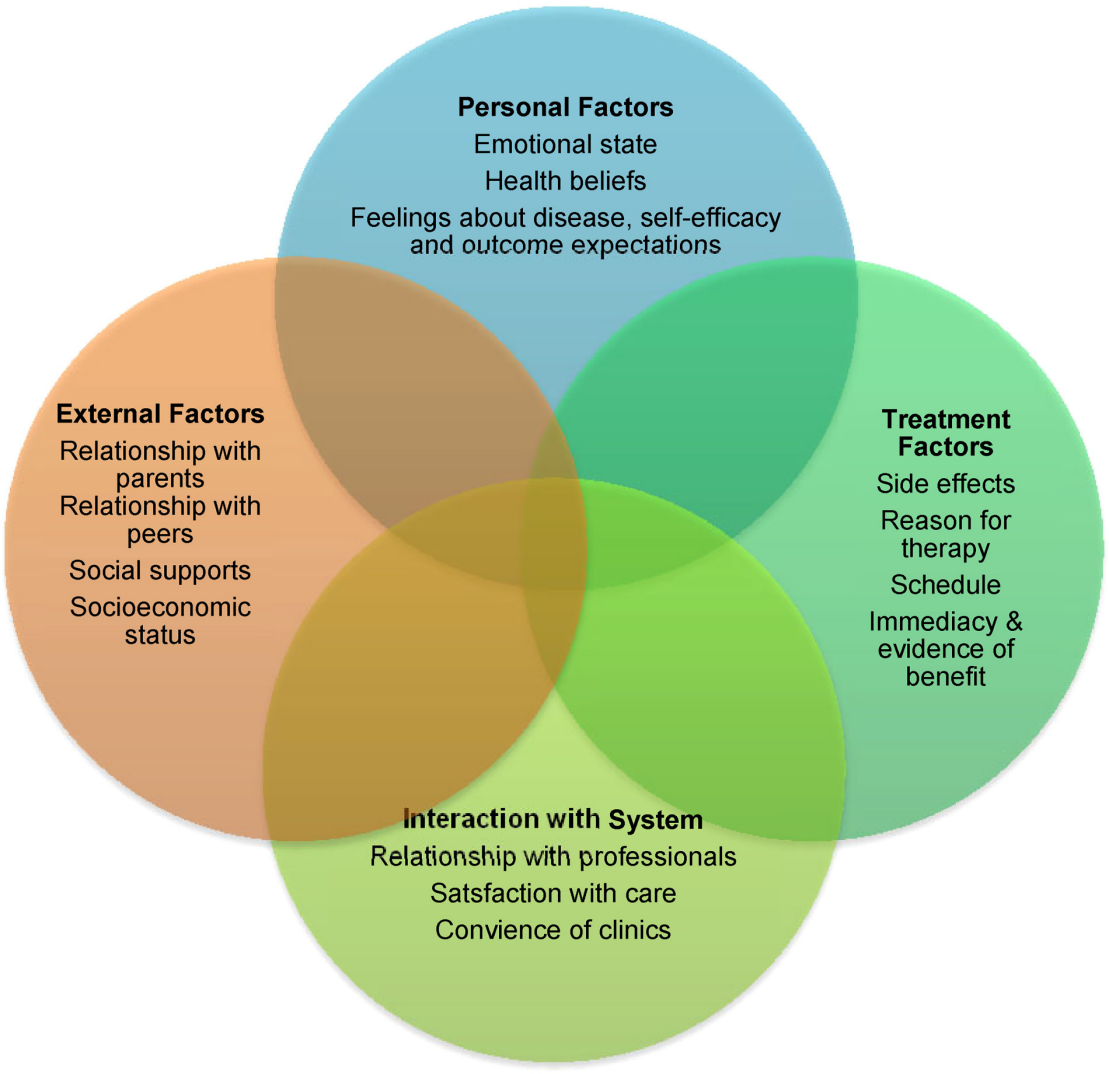
Model of adherence for teenagers and young adults (TYAs) with cancer.

With the factors affecting adherence in mind, Ruddy et al’s. (2009) “model of adherence and persistence” has been adapted to present what could be argued to be a more appropriate theory of adherence for TYAs (Figure 2). Building on the existing theory and the themes that arose from the appraisal, this model was refined using the recommendations/findings from the appraisal to cover all aspects found to affect adherence. When using this four-quadrant approach the personal and external factors, along with treatment factors and interactions with the system should all be considered when addressing adherence.

This model may be useful if implemented into current practice and education to give healthcare professionals an insight into the complexity of the adherence phenomenon. The use of this model could support professionals in recognizing which TYAs may be at great risk of NA. Equally, if NA is identified, the model could also give possible factors that could be affecting adherence for the TYAs, which may give guidance for areas for future support. Through “accurately assessing/identifying the treatment challenges faced by TYAs cancer patients,” healthcare professionals can “facilitate in the elucidation of risk factors and mechanisms underlying patient non adherence” (Kondryn et al., 2009, p. 1330).

### Limitations and Bias

It has become apparent that there is a lack of contemporary, well-designed research in this subject area where there is little primary data. This review has suggested some possible causations for NA in TYAs, but it has become apparent that there needs to be some high-quality research carried out in this area to find ways to help improve adherence. There are several factors that would be interesting to be explored further, for example if there is a difference between genders and adherence in TYAs. This would also be particularly relevant in relation to peer support. Due to a rise in technology and communication being very social media led, the development of an app could potentially be beneficial in addressing adherence.

Due to the small-scale nature of the appraisal only published peer-reviewed journals focussing on adherence were analyzed. The findings not only inform what is already known and how it varies from different studies but also what is not known about this subject area (Hemingway & Brereton, 2009; Nicoll & Beyea, 1999).

Due to the lack of available literature, only one study included in the literature review has been published in the last 5 years. This may cause limitations to the findings as there is a risk that the results of some studies may no longer be relevant. This further supports the need for further research in this area.

## Conclusion

The appraisal highlighted both the complexities and difficulties in measuring adherence, the key factors affecting adherence, and implications for practice. Good communication and relationships are crucial between all parties involved in TYAs’ care including the patients, professionals, parents, and peers. An adapted four-quadrant model has been presented to show the complexity and overlapping factors that affect adherence. It is proposed that this model can act as a useful tool for healthcare professionals caring for TYAs with cancer, as it can serve as a possible indicator for those TYAs who may be likely to become NA, as well as offering some potential causative factors for TYAs who are already NA. It is clear that further research needs to be carried out in this area to find ways to improve adherence, which in turn would increase overall survival.
